# Segond Fracture: From X-ray to Surgical Treatment

**DOI:** 10.7759/cureus.80849

**Published:** 2025-03-19

**Authors:** Hamzah Adwan, Benedikt Wickler, Tobias Hoffmann, Corin Hild, João R Moreira, Detlef Krieg, Boris Bauer, Stefan Rehart, Thomas J Vogl

**Affiliations:** 1 Institute of Radiology, Agaplesion Markus Hospital, Academic Teaching Hospital of the Goethe University, Frankfurt am Main, DEU; 2 Clinic for Radiology and Nuclear Medicine, University Hospital Frankfurt, Goethe University, Frankfurt am Main, DEU; 3 Department of Orthopaedics and Trauma Surgery, Agaplesion Markus Hospital, Academic Teaching Hospital of the Goethe University, Frankfurt am Main, DEU

**Keywords:** arthroscopy, ct, mri, segond fracture, trauma surgery, x-ray

## Abstract

Segond fracture is an avulsion fracture of the lateral side of the tibial plateau. In most cases, this fracture is associated with serious injuries to the knee such as a rupture of the anterior cruciate ligament (ACL). This highlights the importance of recognizing and diagnosing such fractures on X-ray images followed by the use of computed tomography (CT) and magnetic resonance imaging (MRI), in order to accurately diagnose potential additional injuries of the knee joint. This report shows relevant images as well as the outcome of a 59-year-old woman with a right-sided Segond fracture.

## Introduction

Segond fracture represents an avulsion fracture of the proximal tibia at the lateral site of the plateau, which was first described by Dr. Paul Segond (1879) [1]. It is an uncommon fracture [2], with no preference for sex or age [1]. The cause of this fracture is typically an internal rotation of the knee joint combined with varus stress [3].

An accurate diagnosis of Segond fracture is crucial, as it is often associated with additional serious damage of the knee joint such as injuries of the anterior cruciate ligament (ACL) as well as medial collateral ligament (MCL) [4] and meniscus [5]. Various radiological modalities can be applied in the diagnosis of Segond fracture, including X-ray, computed tomography (CT), and magnetic resonance imaging (MRI) [3]. While X-ray and CT are mainly being used to diagnose the fracture itself, MRI should be performed in order to detect potential meniscal and ligamentous injuries of the knee [3].

Patients who practice highly dynamic sports are typically affected. These include soccer, basketball, racket sports, and winter sports [6]. Slagstad et al. showed that only downhill skiing significantly increased the incidence of Segond fracture compared to the rest of the cohort analyzed (p=0.04) [7]. They also describe a mean age of 30 years in their group with Segond fracture, which was significantly older than the non-Segond group, with a mean age of 28 years (p=0.014). The latter had a statistically significantly longer time until surgery from injury at 24 months compared to 16 months in the Segond group (p=0.003) [7].

Segond fractures do not only occur in adults; children and adolescents are also often affected by Segond fractures. The accompanying intra-articular injuries are similar here, but there are small differences, which are assumed to be due to differences in the strength of the bones and ligaments. For example, an increased concomitant occurrence of tibial spine fractures can be observed in children [8].

In this article, we report the case of a 59-year-old woman with a Segond fracture and show diagnostic and treatment-related images as well as her postoperative outcome.

## Case presentation

**Figure 1:**
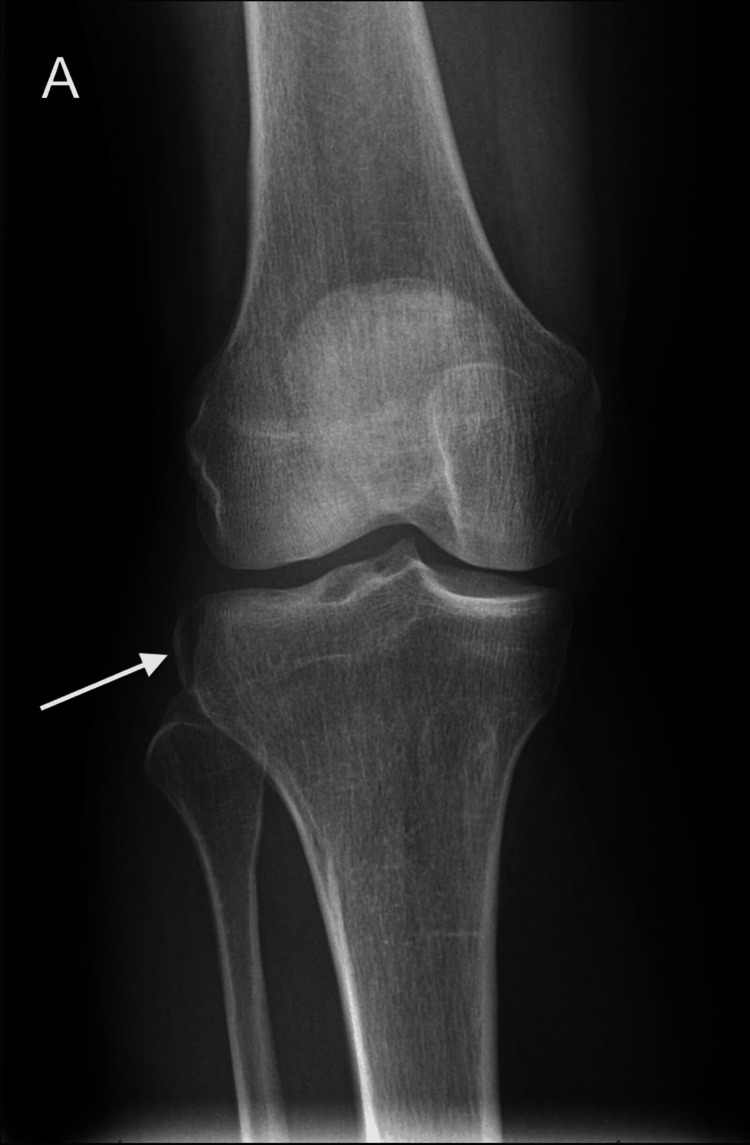
(A) Anterior-posterior X-ray view of the right knee shows an avulsion fracture of the lateral tibial plateau (Segond fracture) with minimal displacement (arrow).

The current report presents the case of a 59-year-old female patient, who initially presented to our emergency department due to excruciating pain in the right knee after a slip injury. The clinical examination revealed a swollen knee and limited range of motion. The peripheral perfusion and motor and sensory functions were intact. Initially, the patient underwent an X-ray imaging (DigitalDiagnost C90, Philips Healthcare, Amsterdam, The Netherlands), as shown in Figure 1.

**Figure 2:**
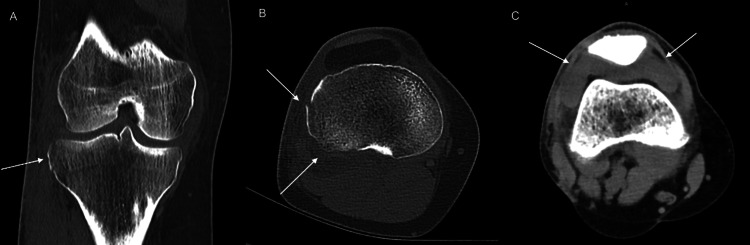
(A) Coronal CT image shows the Segond fracture with a craniocaudal size of 16 mm (arrow). (B) Axial CT image in bone window shows the Segond fracture with a maximum anterior-posterior size of approximately 20 mm as well as cortical incongruence of the dorsolateral tibia plateau as an impression fracture (arrows). The Segond fracture has a minimal lateral displacement. Additional fractures of the knee were ruled out. (C) The axial CT image in soft tissue window shows hemarthrosis in the knee joint (arrows). CT: computed tomography

After the X-ray, the patient underwent a CT scan (SOMATOM Definition AS, Siemens Healthcare, Erlangen, Germany), as shown in Figure 2, in order to demonstrate the fracture more accurately and rule out further bone injuries and fractures.

**Figure 3:**
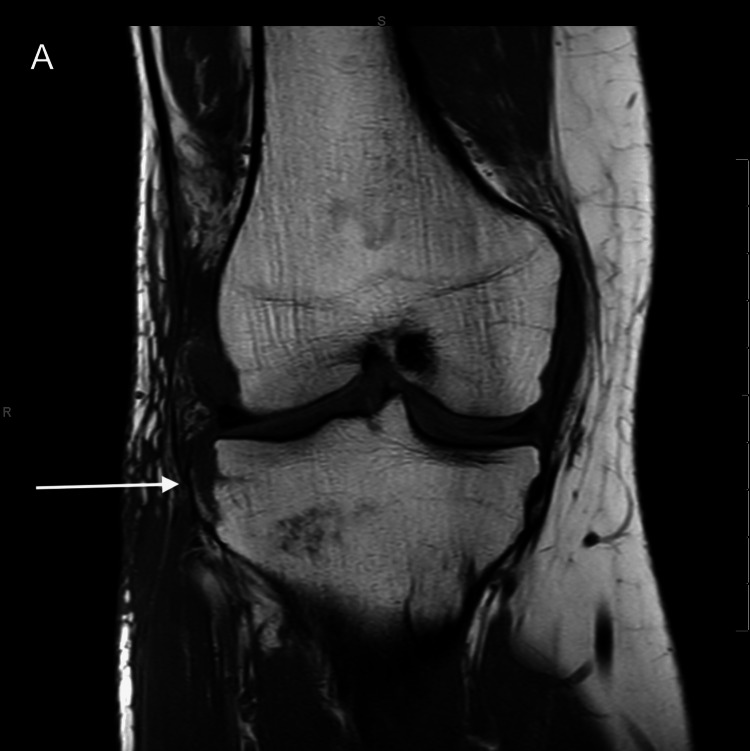
(A) Coronal T1-weighted image shows the Segond fracture (arrow).

**Figure 4:**
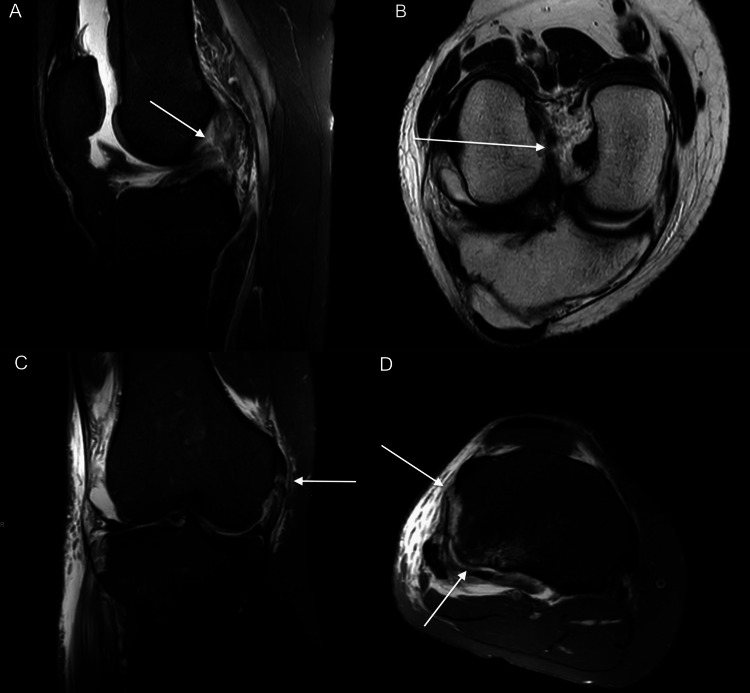
(A) Sagittal fat-suppressed proton density-weighted image and (B) paracoronal T2-weighted image show a total rupture of the ACL (arrows). (C) Coronal fat-suppressed proton density-weighted image demonstrates a partial rupture of the MCL (arrow). (D) Axial fat-suppressed proton density-weighted image shows avulsion of the anterolateral capsular structures involving the anterolateral ligament as well as a posterolateral impression fracture of the tibial plateau (arrows). No injuries to the meniscus were observed. ACL: anterior cruciate ligament, MCL: medial collateral ligament

After confirming the diagnosis of the Segond fracture using CT, an MRI scan (Prodiva 1.5-Tesla, Philips Healthcare, Amsterdam, The Netherlands) was carried out to rule out possible further soft tissue injuries of the knee (Figure 3 and Figure 4).

**Figure 5:**
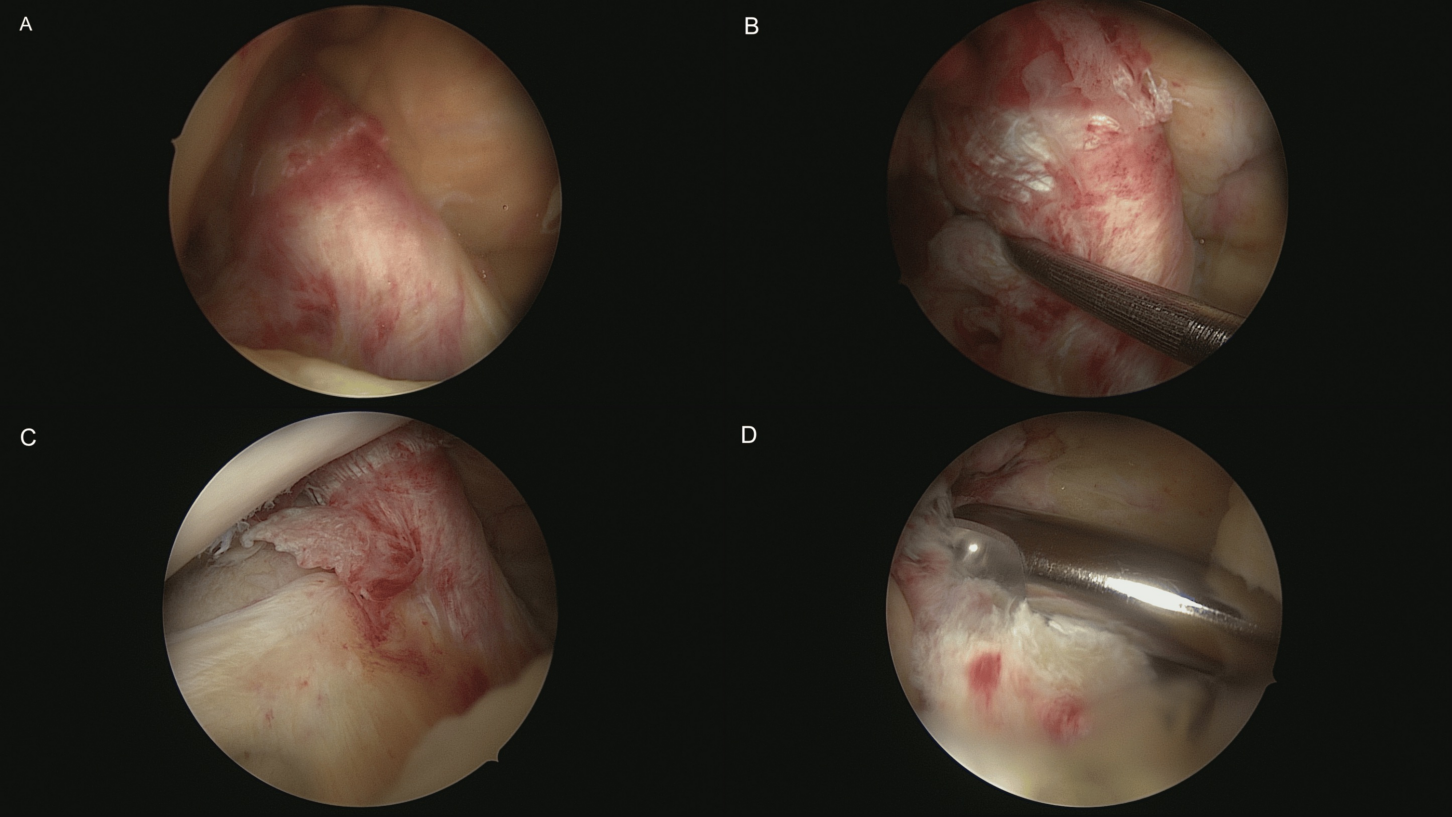
(A-D) The patient initially underwent an arthroscopy of the knee joint, which confirmed the complete rupture of the ACL. The stump of the ACL was then resected. Chondromalacia grade 1 in the notch as well as medial femoral chondromalacia grade 3 were also seen in the arthroscopy. ACL: anterior cruciate ligament

**Figure 6:**
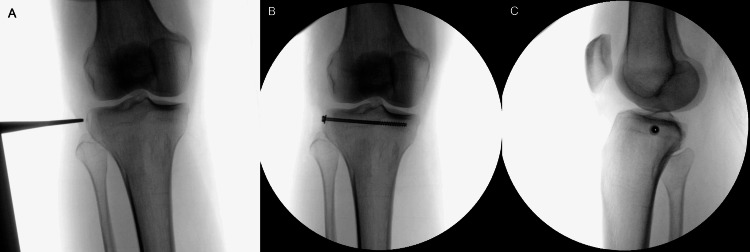
After arthroscopy, the Segond fracture was fixated using a screw: (A) initial intraoperative anterior-posterior view before screwing the Segond fracture and (B) intraoperative anterior-posterior view showing the screw with a length of 6.4 cm and diameter of 0.4 cm. (C) The intraoperative image show the result in lateral view.

**Figure 7:**
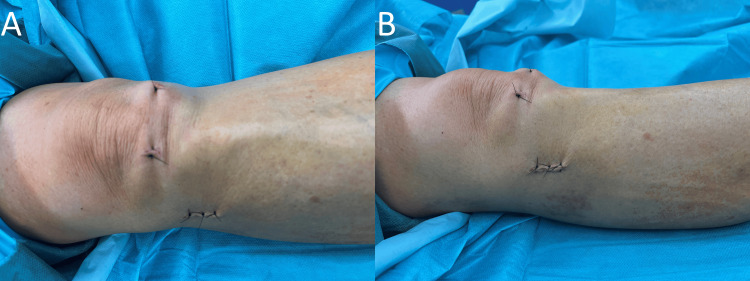
(A and B) Knee after wound closure.

The indication for the surgical treatment of the Segond fracture was given. The benefits and risks were thoroughly discussed with the patient, and she was clarified about possible late complications. Informed consent was obtained, and the patient was surgically treated one week after the injury. The operative images are shown in Figures 5-7.

The surgery went smoothly without complications, and the patient was discharged the day after surgery. Ten weeks after surgery, the patient came for a follow-up. She was doing very well and was able to walk up the stairs. Therefore, a reconstruction of the ACL was not required. However, muscle strengthening was still necessary. Accordingly, the patient did adequate physiotherapy and confirmed that she almost fully recovered without any relevant restrictions or knee instability 28 weeks after the operative treatment, which confirmed again that ACL reconstruction is not needed.

## Discussion

Proximal fractures of the tibia are rare at a rate of 1.2% as shown by Court-Brown et al. in their review of the epidemiology of fractures in adults [9]. Segond fracture represents a tiny avulsion fracture of the proximal tibia [10], which unfortunately may be easily overlooked or misinterpreted due to its size. However, since this seemingly harmless fracture is associated with further serious injuries of the knee joint, a correct and exact diagnosis of this fracture is extremely important for the accurate treatment of patients.

The current report presents all relevant preoperative radiological images including X-ray, CT, and MRI, as well as operative images of a 59-year-old female patient with a Segond fracture. The main associated injuries were a complete rupture of the ACL as well as a partial rupture of the MCL. The fracture was successfully treated with a screw, and the stump of the ACL was resected without complications. The patient was doing well at the follow-up.

Shaikh et al. evaluated in their study the anatomic characteristics of 36 mostly male patients with ACL injuries and Segond fractures [11]. For instance, the reported mean proximal-distal lengths of the fracture were 9.2 mm and 8.7 mm on radiographs and MRI, respectively. However, the craniocaudal size of the fracture in our patient on CT scan was significantly larger at 16 mm. This highlights the fact that Segond fracture is not only heterogeneous regarding associated injuries but also regarding size.

There are no established guidelines for the treatment and management of patients with Segond fracture [1]. It is still under discussion whether a Segond fracture must be surgically treated, as there are studies that show that untreated Segond fractures do not result in increased postoperative instability [12]. Other authors report that the repair of the fracture as well as the ACL results in excellent recovery of the knee's stability [13]. In our case, the fracture was fixated, without the reconstruction of the ACL, but also resulting in a good postoperative outcome without relevant motion restrictions or knee instability 28 weeks postoperatively.

Garra et al. describe in their study from 2024 that the occurrence of concomitant Segond fractures has no significant influence on the rate of return to sport compared to patients with isolated ACL rupture and subsequent surgery and ligament reconstruction [14]. Psychological readiness to return to sport was also analyzed using the ACL-Return to Sport Index and was not found to be significantly different in the study by Garra et al. [14]. These results also indicate that stability in the knee joint can be well restored even in the case of a Segond fracture.

As mentioned, further injuries are associated with Segond fracture besides ACL rupture. Various studies also link them to lateral meniscus damage. In another study by Garra et al., they compared the occurrence of meniscus lesions in patients with ACL injuries. A distinction is made between two groups, a cohort with isolated ACL injury and a group with concomitant Segond fracture. The group with an additional Segond fracture showed a significantly higher incidence of lateral meniscus lesions at 72% compared to the non-Segond group at 49% (p=0.024) [15]. In our case, the patient did not have meniscal injuries.

Sulaiman et al. describe similar findings in their 2021 study with a total of 427 included patients, 12.4% of whom had a Segond fracture, and can also show a significantly increased risk of lateral meniscus lesions when a Segond fracture occurs in addition to the ACL injury (p=0.027) [6].

## Conclusions

In conclusion, it is important to adequately recognize Segond fracture in different imaging modalities, especially on the initial X-ray, as this rare type of fracture is often associated with severe ligamentous and meniscal injuries, requiring appropriate treatment. In our case, fixation of the Segond fracture and performing resection of the ACL stump without reconstruction were effective in the treatment of the current patient's condition.
